# Parasitic zoonoses associated with dogs and cats: a survey of Portuguese pet owners’ awareness and deworming practices

**DOI:** 10.1186/s13071-016-1533-2

**Published:** 2016-05-10

**Authors:** André Pereira, Ângela Martins, Hugo Brancal, Hugo Vilhena, Pedro Silva, Paulo Pimenta, Duarte Diz-Lopes, Nuno Neves, Mónica Coimbra, Ana Catarina Alves, Luís Cardoso, Carla Maia

**Affiliations:** Faculty of Veterinary Medicine, Universidade Lusófona de Humanidades e Tecnologias, Lisboa, Portugal; Hospital Veterinário da Arrábida, Azeitão, Portugal; Clínica Veterinária da Covilhã, Covilhã, Portugal; Faculty of Health Sciences, University of Beira Interior, Covilhã, Portugal; Agrarian College, Polytechnic Institute of Castelo Branco, Castelo Branco, Portugal; Animal and Veterinary Research Centre (CECAV), School of Agrarian and Veterinary Sciences, University of Trás-os-Montes e Alto Douro (UTAD), Vila Real, Portugal; Department of Veterinary Medicine, University School Vasco da Gama, Coimbra, Portugal; Hospital Veterinário do Baixo Vouga, Águeda, Portugal; Amivet – Clínica Veterinária, Évora, Portugal; Hospital Veterinário de Trás-os-Montes, Vila Real, Portugal; VetSantiago – Clínica Veterinária Dr. Duarte Diz-Lopes, Bragança, Portugal; Clube Animal – Centro Veterinário, Beja, Portugal; Clínica Veterinária Porto Seguro, Olhão, Portugal; Global Health and Tropical Medicine, Medical Parasitology Unit, Instituto de Higiene e Medicina Tropical, Universidade Nova de Lisboa, Lisbon, Portugal; Department of Veterinary Sciences, School of Agrarian and Veterinary Sciences, UTAD, Vila Real, Portugal

**Keywords:** Owner awareness, Dogs, Cats, Endoparasites, Ectoparasites, Zoonoses, Portugal

## Abstract

**Background:**

Parasitic diseases of companion animals comprise a group of globally distributed and rapidly spreading illnesses that are caused by a wide range of arthropods, helminths and protozoa. In addition to their veterinary importance, many of these parasites can also affect the human population, due to their zoonotic potential. The aim of the present work was to evaluate the knowledge of Portuguese pet owners regarding the zoonotic potential of parasites that dogs and cats can harbour, most common drugs, frequency of use and reasons for endo- and ectoparasite control.

**Methods:**

Seventy hundred and fifty multiple-choice questionnaires designed to obtain data knowledge about the meaning of zoonosis, knowledge about parasitic diseases and perception regarding their zoonotic potential, as well as the drugs, frequency and reason for deworming their animals were delivered to dog and/or cat owners from non-rural (i.e. urban or semi-urban) and rural parishes who attended veterinary medical centres from continental Portugal.

**Results:**

A total of 536 (71.5 %) questionnaires were retrieved. Two hundred and ninety five (56.5 %) responders had heard of zoonosis/zoonoses, but only 184 (35.2 %) knew their meaning. Tick fever, mange, leishmaniosis and ascaridiosis/roundworms were the parasitic diseases from pets most frequently identified. The number of owners who recognized the different parasitoses, who stated to have heard about zoonoses and who were aware of the potential transmission of parasites from animals to humans was significantly higher in those with intermediate (i.e. ≥9 and ≤ 12 years of schooling) and/or higher academic degree (i.e. licentiate, master’s and/or doctorate degrees). The combinations of febantel-pyrantel-praziquantel (23.5 %) and milbemycin-praziquantel (34.5 %) were the most widely endoparasitic drugs used in dogs and in cats, respectively. The most common ectoparasiticide used in dogs was a combination of imidacloprid-permethrin (33.4 %), while in cats it was imidacloprid (26.3 %) followed by fipronil (25.4 %). The most used treatment schedule against internal and external parasites in dogs and cats was an administration every three months and the main reason to do it was as a prophylactic purpose.

**Conclusions:**

The majority of Portuguese owners that attended veterinarian clinics use endoparasiticides and ectoparasiticides in/on their pets as a prophylactic measure, although in many cases not in the correct schedule of treatment. In addition, most of them are not aware of the possible transmission of parasites from their dogs and cats to themselves, a fact which highlights the important role of veterinarians in the continuous implementation of effective control measures to reduce the risk of parasitic infections in both humans and companion animals.

**Electronic supplementary material:**

The online version of this article (doi:10.1186/s13071-016-1533-2) contains supplementary material, which is available to authorized users.

## Background

Parasitic diseases caused by a wide range of arthropods, helminths and protozoans can cause serious, even life-threatening clinical conditions in dogs and cats, with a number of them also affecting the human population, due to their zoonotic potential, a situation that requires a One Health approach.

Multiple methods and routes of transmission of parasites from domestic carnivores to humans are known. These include transmission via food containing parasite stages (e.g. *Toxoplasma gondii* and *Toxocara* spp.), via water (e.g. *Giardia duodenalis* and *Cryptosporidium parvum*), via direct contact (e.g. *Sarcoptes scabiei*) or percutaneously (e.g. *Ancylostoma* spp.), as well as through one or more vector arthropods (e.g. *Leishmania* spp. and *Dirofilaria* spp.).

Taking into account that dogs and cats in Europe are frequently infected/infested with a wide range of endo- and ectoparasites, including some vector-borne parasites [1–3], the European Scientific Counsel Companion Animal Parasites (ESCCAP; http://www.esccap.org/) has elaborated guidelines for the treatment and control of companion animal parasites, with the aim of protecting not only the health of pets but also the health of the public by reducing the risk of zoonotic parasite transmission. However, awareness of these guidelines and/or the perception of the zoonotic potential of some parasites by pet owners as well as by veterinarians seems to be limited/poor [4–7].

In Portugal, parasitic infections with intestinal helminths (*Ancylostoma* spp., *Dipylidium caninum* and *Toxocara* spp.) and protozoans (*G. duodenalis* and *T. gondii*), as well as fruit fly-borne (*Thelazia callipaeda*), nematocera insect-borne (*Dirofilaria immitis*, *Dirofilaria repens*, *Leishmania infantum*, *Onchocerca lupi*) and tick-borne pathogens (e.g. *Rickettsia conorii*) of zoonotic concern have been reported in domestic and stray dogs and/or cats [1, 8–21]. In addition, human cases of cryptosporidiosis, giardiosis, leishmaniosis, Mediterranean spotted fever (aka tick fever), toxocarosis and toxoplasmosis have also been recorded in the country [12, 22–26].

As information regarding parasite knowledge and control practices in Portugal is limited to only one study made in a referral animal hospital in Lisbon [7], the aim of the present study was to evaluate the knowledge of pet owners from all the five continental Portuguese regions regarding the zoonotic potential of parasites that dogs and cats can harbour as well as the common drugs, frequency and reasons of deworming.

## Methods

### Animals and samples

From October to December 2015, 750 multiple-choice questionnaires were delivered to dog and/or cat owners from non-rural (i.e. urban or semi-urban; with ≥ 2000 habitants) and rural (i.e. with < 2000 habitants) parishes who attended veterinary medical centres from the five continental Portuguese NUTS II (Nomenclature of Units for Territorial Statistics): North, Centre, Alentejo, Lisbon and the Algarve.

The questionnaire was designed in order to obtain the following data: knowledge about the meaning of zoonosis/zoonoses, knowledge about parasitic diseases (i.e. ancylostomatosis, ascaridiosis/roundworms, cryptosporidiosis, dipylidiosis, dirofilariosis/heartworm disease, giardiosis, hydatidosis, leishmaniosis, mange, onchocercosis, thelaziosis, tick fever and toxoplasmosis), and perception regarding the zoonotic potential of parasites their pets can harbour, most common drugs, frequency of use and reason for endo- and ectoparasite control in their pets (Additional file 1, Figure S1).

The commercial names of the different endoparasiticides and ectoparasitices were used in the questionnaire; however, since some of the products share the same active ingredient or association of active ingredients, in the Results and Discussion sections the specific name of each compound (i.e. active ingredient) was used.

### Statistical analysis

Whenever appropriate, the Chi-square and Fisher’s exact tests were used to compare proportions. Pairwise comparisons between categories of the same independent variable incorporated Bonferroni’s correction. A *P*-value < 0.05 was considered as statistically significant. Analyses were performed with SPSS® 21 software for Windows and with StatLib.

## Results

A total of 536 (71.5 %) questionnaires were obtained (141 from North, 132 from Centre, 108 from Lisbon, 118 from Alentejo and 37 from the Algarve), with 56.2 % (294/523) from dog owners, 14.1 % (74/523) from cat owners and 29.6 % (155/523) from responders who had dog and cat as pets. Thirteen responders did not provide information on the pet they owned.

### Knowledge of pet owners about parasitic diseases and awareness of their zoonotic potential

Two hundred and ninety five (56.5 %) of the 522 responders had heard of zoonosis/zoonoses, but only 35.2 % (184/522) knew its meaning (Table 1). The number of owners that stated to have heard about zoonosis and were aware of the potential transmission of parasites from animals to humans was significantly higher in adults (i.e. > 18 and < 65 years old) than in young people (i.e. ≤ 18 years old) and in owners with intermediate (i.e. ≥ 9 and ≤ 12 years of schooling) and higher academic degree (i.e. licentiate, master’s and/ or doctorate degrees) than in the ones with a basic degree (i.e. < 9 years of schooling). There were no differences regarding having heard about and knowing the meaning of zoonosis and the gender, the presence of young members and the place where the responders lived (non-rural or rural parishes).

**Table 1:** Knowledge of the word “zoonosis” and awareness of its meaning by pet owners according to parish of residence, educational qualification, gender, presence of young children in the family and age

Variable/category	Percentage (*n*) of positive responses	
	Zoonoses – has heard of	Zoonoses – understands
Gender	*P* = 0.271	*P* = 0.081
Female	58.5 (193)	38.2 (126)
Male	26.6 (102)	30.2 (58)
Total	56.5 (295)	35.2 (184)
Age (years)	*P* = 0.010	*P* = 0.016
≤ 18	1.0 (1)^a^	0 (0)^b^
19–64	57.6 (279)^a^	36.8 (178)^b^
≥ 65	53.6 (15)	3.3 (6)
Total	56.5 (295)	35.2 (184)
Parish	*P* = 0.232	*P* = 0.094
Rural	52.4 (89)	30.0 (51)
Non-rural	58.3 (203)	37.9 (132)
Total	56.4 (292)	35.3 (183)
Schooling (degree)	*P* < 0.001	*P* < 0.001
Basic *	27.3 (12)^c^	11.4 (5)^e,f^
Intermediate **	44.8 (78)^d^	18.4 (32)^e,g^
Academic ***	66.4 (188)^c,d^	46.3 (131)^f,g^
Total	55.5 (278)	33.5 (168)
Presence of young members	*P* = 0.811	*P* = 0.110
Yes	57.8 (74)	41.4 (53)
No	56.1 (221)	33.2 (131)
Total	56.6 (295)	64.8 (238)

* < 9 years of schooling; ** ≥ 9 and ≤ 12 years of schooling; *** licentiate, master’s and/ or doctorate degrees. Bonferroni correction (i.e. multiplying each *P*-value by 3) has been incorporated between categories: ^a^ *P* = 0.003; ^b^ *P* = 0.048; ^c^ *P* < 0.001; ^d^ *P* < 0.001; ^e^ *P* = 1.0; ^f^ *P* < 0.001; ^g^ *P* < 0.001. Only statistically significant differences are shown

Regarding the recognition of parasitic diseases from pets, the most frequently identified were tick fever, mange, leishmaniosis and ascaridiosis/roudworms (Table 2). There was a significant difference between the percentage of owners that identified dirofilariosis and toxoplasmosis, hydatidosis and dirofilariosis, and giardiosis and hydatidosis as parasitic diseases of companion animals (Table 2). Less than 15 % of the responders identified dipylidiosis, cryptosporidiosis, ancylostomosis, onchocercosis and thelaziosis as parasitic diseases of dogs and cats (Table 2).

**Table 2:** Identification of parasitic diseases of dogs and cats by the responders

Disease	Awareness/respondents (*n*)	Frequency (%)
Tick fever	524/531	98.7
Mange	519/531	97.7
Leishmaniosis	484/530	91.3
Ascaridiosis/roundworms	481/531	90.6
Toxoplasmosis	369/531	69.5^a^
Dirofilariosis/heartworm disease	235/478	49.2^a^
Hydatidosis	210/530	39.6^a^
Giardiosis	131/531	24.7^a^
Dipylidiosis	74/508	14.6
Cryptosporidiosis	70/530	13.2
Ancylostomatosis	69/531	13.0
Onchocercosis	65/530	12.3
Thelaziosis	58/531	10.9

^a^Statistically significant difference to the frequency value positioned immediately above

Furthermore, leishmaniosis was recognised by a significantly high number of owners that lived in non-rural parishes than the ones that lived in rural areas (Table 3). Identification of cryptosporidiosis, giardiosis, hydatidosis, leishmaniosis, mange and toxoplasmosis was significantly higher in the owners with an academic degree than in the responders with basic and intermediate educational qualifications, while the recognition of ancylostomosis, onchocercosis and thelaziosis was significantly lower in the responders with an intermediate degree than in the ones with academic qualifications.

**Table 3:** Identification of common parasitic diseases by owners of dogs and cats

Variable/category	Percentage (*n*) of positive responses – “Have you ever heard of?”												
	Ancylostomatosis	Ascaridiosis	Cryptosporidiosis	Dipylidiosis	Dirofilariosis	Tick fever	Giardiosis	Hydatidosis	Leishmaniosis	Onchocercosis	Mange	Thelaziosis	Toxoplasmosis
Parish	*P* = 1.0	*P* = 0.816	*P* = 0.610	*P* = 0.652	*P* = 0.911	*P* = 0.902	*P* = 1.0	*P* = 0.945	*P* = 0.003	*P* = 0.954	*P* = 0.747	*P* = 0.245	*P* = 0.969
Rural	12.9 (23)	89.9 (160)	11.9 (21)	13.3 (23)	48.5 (82)	98.3 (175)	24.7 (44)	40.1 (71)	85.9 (152)	11.9 (21)	98.3 (175)	8.4 (15)	69.1 (123)
Non-rural	13.0 (46)	90.9 (321)	13.9 (49)	15.2 (51)	49.5 (153)	98.9 (349)	24.6 (87)	39.4 (139)	94.1 (332)	12.5 (44)	97.5 (344)	12.2 (43)	69.7 (246)
Total	13.0 (69)	90.6 (481)	13.2 (70)	14.6 (74)	49.2 (235)	98.7 (524)	24.7 (131)	39.6 (210)	91.3 (484)	12.3 (65)	97.7 (519)	10.9 (58)	69.5 (369)
Schooling (degree)	*P* < 0.001	*P* = 0.076	*P* < 0.001	*P* = 0.624	*P* = 0.242	*P* = 0.225	*P* < 0.001	*P* < 0.001	*P* < 0.001	*P* < 0.001	*P* = 0.040*	*P* < 0.001	*P* < 0.001
Basic	10.4 (5)	89.6 (43)	4.2 (2)^b^	17.0 (8)	37.8 (17)	97.9 (47)	14.6 (7)^d^	22.9 (11)^f^	70.8 (34)^h,i^	4.2 (2)	95.8 (46)	10.4 (5)	43.8 (21)^m^
Intermediate	3.3 (6)^a^	87.2 (157)	3.9 (7)^c^	15.8 (27)	50.6 (81)	100 (180)	12.8 (23)^e^	28.5 (51)^g^	89.4 (160)^h,j^	7.3 (13)^k^	96.1 (173)	2.2 (5)^l^	57.8 (104)^n^
Academic	20.2 (58)^a^	93.4 (268)	21.7 (62)^b,c^	13.0 (36)	51.2 (132)	99.3 (285)	34.5 (99)^d,e^	50.5 (145)^f,g^	95.8 (275)^I,j^	18.5 (53)^k^	99.3 (285)	17.1 (49)^l^	82.6 (237)^m,n^
Total	13.4 (69)	90.9 (468)	13.8 (71)	14.4 (71)	49.7 (230)	99.4 (512)	25.0 (129)	40.3 (207)	91.2 (469)	13.2 (68)	97.9 (504)	11.3 (58)	70.3 (362)

Bonferroni correction (i.e. multiplying each *P* value by 3) has been incorporated between schooling categories, and only statistically significant differences are shown; ^a^ *P* < 0.001; ^b^ *P* = 0.024; ^c^ *P* < 0.001; ^d^ *P* = 0.030; ^e^ *P* < 0.001; ^f^ *P* = 0.003; ^g^ *P* < 0.001; ^h^ *P* = 0.009; ^i^ *P* < 0.001; ^j^ *P* = 0.036; ^k^ *P* = 0.003; ^l^ *P* < 0.001; ^m^ *P* < 0.001; ^n^ *P* = 0.024; *Statistically significant difference(s) not confirmed after pairwise comparisons

The awareness of the zoonotic potential of ascaridiosis, hydatidosis, mange and toxoplasmosis among the responders who had previously heard of them was significantly higher in the owners with an academic degree than in the ones with an intermediate level of education (Table 4).

**Table 4:** Awareness of the potential zoonotic character of each particular disease among those responders who have previously heard of them

Variable/category	Percentage (n) of positive responses – “Are you aware of the zoonotic character of?”												
	Ancylostomatosis	Ascaridiosis	Cryptosporidiosis	Dipylidiosis	Dirofilariosis	Tick fever	Giardiosis	Hydatidosis	Leishmaniosis	Onchocercosis	Mange	Thelaziosis	Toxoplasmosis
Parish	*P* = 0.100	*P* = 0.157	*P* = 0.739	*P* = 0.377	*P* = 0.918	*P* = 0.872	*P* = 1.0	*P* = 0.741	*P* = 0.220	*P* = 0.243	*P* = 0.220	*P* = 0.334	*P* = 0.135
Rural	56.5 (13)	66.5 (105)	61.9 (13)	52.2 (12)	19.8 (16)	74.4 (128)	60.5 (26)	54.9 (39)	34.2 (51)	33.3 (7)	71.7 (124)	33.3 (5)	64.8 (79)
Non-rural	32.6 (15)	73.2 (232)	54.2 (26)	38.0 (19)	18.2 (27)	75.5 (262)	61.2 (52)	51.4 (71)	40.6 (134)	52.3 (23)	77.1 (262)	52.4 (22)	73.0 (178)
Total	40.6 (28)	70.9 (337)	56.5 (39)	42.5 (31)	18.8 (43)	75.1 (390)	60.9 (78)	52.6 (110)	38.6 (185)	46.2 (30)	75.2 (386)	47.4 (27)	70.2 (257)
Schooling (degree)	ND*	*P* = 0.013	ND*	ND*	*P* = 0.109	*P* = 0.963	ND*	*P* = 0.022	*P* = 0.083	ND*	*P* = 0.025	ND*	*P* < 0.001
Basic	60.9 (3)	73.2 (30)	50.0 (1)	50.0 (4)	29.4 (5)	73.3 (33)	66.7 (4)	27.3 (3)	32.4 (11)	50.0 (1)	79.5 (35)	80.0 (4)	66.7 (14)
Intermediate	16.7 (1)	61.3 (95)^a^	28.6 (2)	29.6 (8)	23.8 (19)	75.3 (134)	36.4 (8)^b^	40.0 (20)	30.4 (48)	7.7 (1)^c^	67.8 (116)^d^	0 (0)	52.4 (54)^e^
Academic	41.4 (24)	74.8 (199)^a^	59.0 (36)	54.3 (19)	14.1 (18)	74.6 (212)	67.3 (66)^b^	57.9 (84)	40.8 (28)	52.8 (28)^c^	78.8 (223)^d^	47.9 (23)	76.6 (180)^e^
Total	40.6 (28)	70.1 (324)	55.7 (39)	44.4 (31)	18.7 (42)	74.8 (379)	61.9 (78)	51.9 (107)	36.6 (30)	44.1 (30)	75.1 (374)	47.4 (27)	69.1 (248)

Bonferroni correction (i.e. multiplying each *P*-value by 3) has been incorporated between schooling categories, and only statistically significant differences are shown; ^a^ *P* = 0.015; ^b^ *P* = 0.042; ^c^ *P* = 0.027; ^d^ *P* = 0.039; ^e^ *P* < 0.001; *ND* not validated; *Statistically significant difference(s) not confirmed after pairwise comparisons

### Endoparasitic and ectoparasitic drugs, frequency and reason for deworming

At the time of the study (i.e. between October and December 2015), 95.5 % (429/449) of the dogs and 90.7 % (205/226) of the cats were being treated with endoparasiticide drugs, while 98.2 % (440/448) of the dogs and 91.7 % (209/228) of the cats were treated with ectoparasiticides.

The most widely endoparasitic drug used in dogs (23.5 %) was a combination of febantel-pyrantel-praziquantel, while in cats (34.5 %) it was a combination of milbemycin-praziquantel (Table 5). In addition, 17.6 and 22.3 % of the owners did not know the drug they used to deworm their dogs and cats, respectively. Regarding frequency of deworming, 33.7 % of the dog owners dewormed every three months and 27.6 % every four months. Only 6.4 % used endoparasiticides monthly. In a similar manner to the dog owners, 38.2 % (78/204) of the cat owners dewormed their cats against internal parasites every three months, and 30.9 % (63/204) every four months. Only 2 % of the cat owners dewormed their animals against internal parasites monthly.

**Table 5:** Endoparasiticides and ectoparasiticides used in/on dogs and cats

Product	Dogs	Cats
	No. (%)	No. (%)
Endoparasiticides	545	220
Emodepside + praziquantel	41 (7.5)	8 (3.6)
Emodepside + toltrazuril	5 (0.9)	0 (0)
Eprinomectin + fipronil + praziquantel + s-methoprene	2 (0.4)^a^	9 (4.1)
Epsirantel + pyrantel	3 (0.6)	7 (3.2)
Febantel + praziquantel + pyrantel	128 (23.5)	2 (0.9)
Febantel + pyrantel	18 (3.3)	0 (0)
Fenbendazole	1 (0.2)	2 (0.9)
Fenbendazole + praziquantel	18 (3.3)	0 (0)
Ivermectin	5 (0.9)	0 (0)
Ivermectin + pyrantel	8 (1.5)	0 (0)
Mebendazole	5 (0.9)	1 (0.5)
Moxidectin	1 (0.2)	0 (0)
Mylbemicine + praziquantel	98 (18.0)	76 (34.5)
Niclosamide + oxibendazole	6 (1.1)	3 (1.4)
Nitroscanate	4 (0.7)	0 (0)
Oxibendazole + praziquantel	4 (0.7)	0 (0)
Praziquantel	23 (4.2)	2 (0.9)
Praziquantel + pyrantel	66 (12.1)	45 (20.5)
Pyrantel	13 (2.4)	13 (5.9)
Selamectin	0 (0)	3 (1.4)
Do not know	96 (17.6)	49 (22.3)
Ectoparasiticides	625	236
Afoxalaner	8 (1.3)	0 (0)
Amitraz	1 (0.2)	0 (0)
Deltamethrin	87 (13.9)	0 (0)
Dinotefuran + permethrin + pyriproxyfen	18 (2.9)	0 (0)
Dinotefuran + pyriproxyfen	0 (0)	3 (1.3)
Eprinomectin + fipronil + praziquantel + s-methoprene	2 (0.3)	17 (7.2)
Fipronil	63 (10.1)	60 (25.4)
Fipronil + permethrin	11 (1.8)	0 (0)
Fipronil + s-methoprene	28 (4.5)	23 (9.7)
Flumethrin + imidacloprid	39 (6.2)	13 (5.5)
Fluralaner	38 (6.1)	0 (0)
Imidacloprid	36 (5.8)	62 (26.3)
Imidacloprid + moxidectin	13 (2.1)	18 (7.6)
Imidacloprid + permethrin	209 (33.4)	0 (0)
Indoxacarb	17 (2.7)	15 (6.4)
Lufenuron	6 (1.0)	0 (0)
Permethrin	24 (3.8)	1 (0.4)^a^
Pyriprole	1 (0.2)	0 (0)
Selamectin	6 (1.0)	3 (1.3)
Spinosad	0 (0)	3 (1.3)
Do not know	18 (2.9)	18 (7.6)

^a^Although the product is not approved for this species, pet owners have declared to apply it

Regarding the use of ectoparasiticides in dogs, the most widely used was a combination of imidacloprid-permethrin (33.4 %), while in cats imidacloprid (26.3 %) followed by fipronil (25.4 %) were the most common ones. Eighteen (2.9 %) of the dog owners and 18 (7.6 %) of the cat owners did not know the product used against external parasites (Table 5). One cat owner (0.4 %) used permethrin as ectoparasiticide.

One hundred and twenty-six (29.2 %) out of 431 dog owners treated their animals every three months against ectoparasites, and 124 (28.8 %) monthly. Thirty (7.0 %) out of 431 dog owners treated their animals yearly. Regarding cats, 56 (28 %) out of 200 owners treated their animals with ectoparasiticides every three months and 26 % (52/200) monthly. Only 19 (9.5 %) of the cat owners used ectoparasiticides every four months.

The main reason to use endo- and ectoparasiticides in dogs (82.1 %) and cats (83.2 %) was as a prophylactic purpose, while 3.7 % of the dog owners and 3.9 % of the cat owners gave antiparasitic products as a treatment for a previously diagnosed/suspected parasitic infection/infestation. About 13.0 % of dog and 12.5 % of cat owners dewormed their pets following recommendation by the veterinarian, and a minor percentage (1.2 %) did it for other reasons (e.g. public health concern).

## Discussion

Given the clinical importance of intestinal parasites and vector-borne pathogens affecting pets, their global presence and the zoonotic impact of many of them, public education is crucial for reducing risk exposure in both humans and companion animals [3, 27]. In Portugal intestinal helminths and protozoans, as well as vector-borne parasites, have been reported in domestic and stray dogs and/or cats [8–21, 28–31], with some of them having also been recorded in humans [12, 22–26]. In the present study 56.5 % of the responders had heard of the word “zoonosis” and 35.2 % of them (46.3 % among people with an academic degree), were aware of the possible transmission of parasites from their pets to themselves, which was higher than the 29.8 % obtained in a previous study made in São Paulo state (Brazil) [32] but lower than the 49.2 and 75.0 % obtained from pet owners from northern Italy [6] or from a referral small animal hospital in Lisbon, Portugal [7], respectively. The differences regarding the perception of human health risks due to canine and feline parasites among the Portuguese surveys might be related with the fact that the present one was a written questionnaire without an oral interview that could have potentially helped owners to better express their concept of zoonosis. Furthermore, the current investigation was carried out with pet owners that for several reasons attended veterinary clinics/hospitals outside Lisbon (i.e. the capital city), contrarily to the one performed by Matos et al. [7], which comprised owners that visited a referral hospital in Lisbon for a second opinion/specialist appointment, and that were probably more well informed of the health hazards associated with the ownership of a pet. In the same line of thought, this might be the reason why in the present study adult pet owners and the ones with an intermediate or academic degree were more aware of the potential transmission of parasites from animals to humans. In fact, the low schooling level of owners has been regarded as a risk factor for the occurrence of zoonotic gastrointestinal parasites (*Ancylostoma* spp., *Cryptosporidium* spp., *G. duodenalis*, *T. gondii* and *Toxocara* spp.) in dogs [33] and in cats [34].

Tick fever, mange, leishmaniosis and ascaridiosis/roudworms were the parasitic and/or arthropod/vector-borne diseases most frequently identified by the responders to the present survey, a situation that might be related with the high frequency of roundworms [8, 15, 16, 30] and leishmaniosis [11, 13] reported in Portuguese dogs and cats, as well as with the association of tick fever (i.e. Mediterranean spotted-fever [35]) and sarcoptic mange with human diseases. The differences between the number of pet owners who identified dirofilariosis, toxoplasmosis, hydatidosis and giardiosis might be related with the geographical region where they lived as well as with gender since canine heartworm disease is more common in the Centre and in the South of Portugal [11, 36], most of the reported cases of human hydatidosis are notified from Alentejo [37], giardiosis has been reported in children from Lisbon [24, 38], and toxoplasmosis is mostly associated with pregnancy or women of childbearing age [39]. The fact that less than 15 % of the responders identified ancylostomosis, cryptosporidiosis, dipylidiosis, onchocercosis and thelaziosis as parasitic diseases of the dogs and cats, might be related with the difficulty in observing the first two parasites in the faeces of their pets due to the small size of *Ancylostoma* spp. adult parasites (contrarily to *Toxocara* spp.) and *Cryptosporidium* spp. oocysts, to the misidentification of *D. caninum* with roundworms, and to the fact that the detection of the last two nematodes is relatively recent in Portugal [40, 41].

According to the ESCCAP guidelines and depending on the different scenarios (e.g. presence of children or outdoor access), the frequency of treatment against internal parasites should be at least four times per year, at intervals not exceeding three months apart or preferably a monthly treatment, while treatment against ectoparasites should also be done monthly. In the present study, 67.7 % (285/421) of the dog owners and 71.1 % (145/204) of the cat owners treated their pets against endoparasites at every four, three or one months. When a year-round control is not performed (in the present study internal monthly deworming was only performed by 6.4 % of the dog owners and 2 % of the cat owners) veterinarians should inform owners about the importance of regular faecal examinations (monthly or at least quarterly) for evaluating the (re)occurrence of intestinal parasites (www.esccap.org). In the same line, and since only 26 % of the dog owners and 28.8 % of the cat owners monthly treated their pets against ectoparasites, it would also be important to screen companion animals for vector-borne pathogens (e.g. *L. infantum* and *D. immitis*) at least once a year, preferably outside the period of vector activity, as this procedure would probably contribute to more detection of established infections.

The endoparasiticides most commonly used in the dogs (i.e. febantel-pyrantel-praziquantel, and milbemycin-praziquantel) and in the cats (i.e. milbemycin-praziquantel, and pyrantel-praziquantel) from the present study are effective against roundworms, hookworms, tapeworms and heartworms (only the ones containing mylbemycin, against heartworms). Therefore, these pets can be considered protected from these helminths as long as the owners comply with the correct schedule of treatment; however, these drugs are not effective against intestinal protozoa, which is probably the reason why *Cystoisospora* spp., *G. duodenalis* and *T. gondii* infections are quite common in domestic dogs and cats from Portugal [10, 15, 16, 42, 43]. On the other hand, the reason why vector-borne pathogens are quite frequent in Portuguese domestic dogs and cats [11, 13, 17, 29, 44], even when they are treated with effective ectoparasiticide products against vector arthropods (e.g. imidacloprid-permethrin for dogs and imidacloprid and fipronil for cats) is probably related with the low percentage of animals protected year-round or, as referred for anthelminthics, to the lack of compliance for ectoparasiticide application, or in the case of cats to the impossibility to apply pyrethroids (the only repellents effective against sand flies), due to their toxicity to felids.

## Conclusions

The majority of the Portuguese pet owners that attend veterinary clinics administrate endoparasiticides and ectoparasitices to their pets as a prophylatic measure against internal and external parasites, although in many cases not in the correct schedule of treatment. In addition, the present study highlights the important role of veterinarians in providing owners with concise but objective information about the ways of pathogen transmission to their pets and themselves, which can be done for instance through appellative and easy-to-understand flyers, regular newsletters using social networks, and free or low cost workshops of short duration. The increase of owners’ awareness about zoonotic and parasitic diseases should also include the implementation of effective control measures to reduce the risk of parasitic infections in both companion animals and humans, which can be done by reminding, via online or short message service, about the importance of faecal microscopic examinations and screening of vector-borne pathogens in a regular basis, as well as through accurate deworming schedules and appropriate life-long actions and regular removal and disposal of faeces to minimize environmental contamination.

## Additional file

Additional file 1: Figure S1. Questionnarie regarding dog and cat owners awareness on parasitic diseases, their zoonotic potential and the endo- and ectoparasiticide products most commonly used, frequency and reason of administration to their pets. (DOCX 82 kb)
