# Supplementary material for: Parasitic zoonoses associated with dogs and cats: a survey of Portuguese pet owners’ awareness and deworming practices
Source: Parasit Vectors. 2016 May 10;9:245. doi: 10.1186/s13071-016-1533-2 (PMC4862121; doi:10.1186/s13071-016-1533-2)
Supplement: Additional file 1: Figure S1. — Questionnarie regarding dog and cat owners awareness on parasitic diseases, their zoonotic potential and the endo- and ectoparasiticide products most commonly used, frequency and reason of administration to their pets. (DOCX 82 kb) [file 13071_2016_1533_MOESM1_ESM.docx]

Additional file 1: Figure S1

Questionnarie regarding dog and cat owners awareness on parasitic diseases, their zoonotic potential and the endo- and ectoparasiticide products most commonly used, frequency and reason of administration to their pets.

**Information related to the owner**

**Age:** ______ years

**Schooling (degree):**

| - Basic |
| --- |
| - Intermediate |
| - Academic |

**To your household belong:**

| - Young people (age ≤18 years) |
| --- |
| - Adults (age >18 years and <65 years) - Seniors (age ≥65 years) |

| **Gender:** | - F | - M |
| --- | --- | --- |

**Parish:**

**County:**

**Regarding the word ZOONOSIS:**

| - Yes, I know its meaning |
| --- |
| - I’ve heard but I don’t know what it means |
| - No, I don´t know that word |

**Do you think that INTERNAL parasites of dog/cat can be transmitted to human beings?**

| - Yes | - No | - I don´t know |
| --- | --- | --- |

dd

**Do you think that EXTERNAL parasites of dog/cat can be transmitted to human being?**

| - Yes | - No | - I don´t know |
| --- | --- | --- |

**With regard to the following diseases:**

|  | Have you heard of it? | | **IF YES**, do you think it’s transmissible to humans? | | | |
| --- | --- | --- | --- | --- | --- | --- |
| **Ancylostomatosis** | - Yes | - No | - Yes | - No | - I don’t know |  |
| **Ascaridiosis/roundworms** | - Yes | - No | - Yes | - No | - I don’t know |  |
| **Cryptosporidiosis** | - Yes | - No | - Yes | - No | - I don’t know |  |
| **Dipylidiosis** | - Yes | - No | - Yes | - No | - I don’t know |  |
| **Dirofilariosis/heartworm disease** | - Yes | - No | - Yes | - No | - I don’t know |  |
| **Giardiosis** | - Yes | - No | - Yes | - No | - I don’t know |  |
| **Hydatidosis** | - Yes | - No | - Yes | - No | - I don’t know |  |
| **Leishmaniosis** | - Yes | - No | - Yes | - No | - I don’t know |  |
| **Mange** | - Yes | - No | - Yes | - No | - I don’t know |  |
| **Onchocercosis** | - Yes | - No | - Yes | - No | - I don’t know |  |
| **Thelaziosis** | - Yes | - No | - Yes | - No | - I don’t know |  |
| **Tick fever/rickettsiosis** | - Yes | - No | - Yes | - No | - I don’t know |  |
| **Toxoplasmosis** | - Yes | - No | - Yes | - No | - I don’t know |  |

| **Gender:** |
| --- |

**Please, fill in only if you have a cat** – Information related to your(s) cat(s)

|  | Number of females _____ | Number of males_____ |
| --- | --- | --- |
|  |  |  |

| **Do you deworm your cat(s) against external parasites?** | - Yes | - No |
| --- | --- | --- |

**If yes, with which frequency?**

|  | - Once per month | - 3–3 months | - 4–4 months | - 6–6 months | - Once per year |
| --- | --- | --- | --- | --- | --- |

- Other. Which one?_______________________________________

**Which product(s) do you usually use?**

| - Activyl® |
| --- |
| - Advantage® - Advocate® - Broadline® - Capstar® - Frontline® - Frontline Combo® |
|  |

- Program®
- Seresto®
- Vectra Felis®
- Other. Which one?____________________________
- I don’t know

| **Do you deworm your cat(s) against internal parasites?** | - Yes | - No |
| --- | --- | --- |

**If yes, with which frequency?**

|  | - Once per month | - 3–3 months | - 4–4 months | - 6–6 months | - Once per year |
| --- | --- | --- | --- | --- | --- |

- Other. Which one?_______________________________________

**Which product(s) do you usually use?**

| - Broadline® - Dosalid® |
| --- |
| - Drontal® - Milbemax® - Panacur® - Profender® - Strongid® |
|  |

- Telmin®
- Vitaminthe®
- Zipyran®
- Other. Which one? ____________________________
- I don’t known

**Why do you deworm your cat(s)?**

| - To prevent them from getting parasites. |
| --- |
| - As a treatment, when I detect parasites. - Because my veterinarian recommends me to do so. - Other reason. Which one?____________________________________________________ |
|  |

| **Gender:** |  |
| --- | --- |

**Please, fill in only if you have a dog** – Information related to your(s) dog(s)

|  | Number of females _____ | Number of males_____ |
| --- | --- | --- |
|  |  |  |

| **Do you deworm your dog(s) against external parasites?** | - Yes | - No |
| --- | --- | --- |

**If yes, with which frequency?**

|  | - Once per month | - 3–3 months | - 4–4 months | - 6–6 months | - Once per year |
| --- | --- | --- | --- | --- | --- |

- Other. Which one?_______________________________________

**Which product (s) do you usually use?**

| - Activyl® |
| --- |
| - Advantage® - Advantix® - Advocate® - Bravecto® - Effitix® - Frontline® - Frontline Combo® - Frontline Tri-act® |
|  |

- Nexgard®
- Program®
- Pulvex®
- Scalibor®
- Seresto®
- Stronghold®
- Vectra 3D®
- Other. Which one?____________________________
- I don’t know

| **Do you deworm your dog(s) against internal parasites?** | - Yes | - No |
| --- | --- | --- |

**If yes, with which frequency?**

|  | - Once per month | - 3–3 months | - 4–4 months | - 6–6 months | - Once per year |
| --- | --- | --- | --- | --- | --- |

- Other. Which one?_______________________________________

**Which product(s) do you usually use?**

| - Caniquantel plus® - Dolpac® |
| --- |
| - Drontal® - Drontal plus® - Heartgard 30 - Heartgard 30 plus® - Lopatol® - Milbemax® - Profender® |
|  |

- Procox®
- Strongid®
- Telmin®
- Vitaminthe®
- Zipyran®
- Zipyran plus®
- Other. Which one? ____________________________
- I don’t Known

**Why do you deworm your dog(s)?**

| - To prevent them from getting parasites. |
| --- |
| - As a treatment, when I detect parasites. - Because my veterinarian recomends me to do so. - Other reason. Which one?____________________________________________________ |
|  |

**THANK YOU VERY MUCH! internamente o seu animal?**
